# Autoimmune Hypothyroidism Presenting as Subacute Intestinal Obstruction: A Case Report and Literature Review

**DOI:** 10.7759/cureus.114214

**Published:** 2026-08-09

**Authors:** Eman Ahmed, Mandeep Singh, Khalid Abdur Rashid

**Affiliations:** 1 Internal Medicine, Emirates Health Services, Sharjah, ARE; 2 General Internal Medicine, Princess Alexandra Hospital National Health Service (NHS) Trust, Harlow, GBR

**Keywords:** autoimmune, diabetic ketoacidosis (dka), hypokalemia related medical emergencies, hypothyroid, intestinal pseudo-obstruction

## Abstract

Hypothyroidism is associated with multiple gastrointestinal manifestations, ranging from mild constipation to rare life-threatening complications such as paralytic ileus and intestinal pseudo-obstruction. We report a case of a 41-year-old male patient with severe autoimmune hypothyroidism presenting with persistent abdominal distension and subacute intestinal obstruction secondary to fecaloma and colonic ileus. The diagnosis was initially obscured by concomitant diabetic ketoacidosis (DKA) and electrolyte disturbances. Despite resolution of DKA and multiple bowel decompression procedures, gastrointestinal symptoms persisted until hypothyroidism was recognised and treated with levothyroxine. This case highlights the importance of considering endocrine causes, particularly severe hypothyroidism, in patients with unexplained intestinal dysmotility and bowel obstruction.

## Introduction

Hypothyroidism is a common endocrine disorder resulting from deficient thyroid hormone production and is associated with a wide range of systemic manifestations, including fatigue, weight gain, cold intolerance, dry skin, and constipation. Gastrointestinal involvement is common and includes delayed gastric emptying, reduced intestinal transit, abdominal bloating, and constipation, reflecting the effects of thyroid hormone deficiency on gastrointestinal motility. In rare cases, profound gastrointestinal hypomotility may progress to paralytic ileus, fecal impaction, intestinal pseudo-obstruction, or even megacolon [1].

The underlying pathophysiology involves reduced autonomic stimulation, impaired smooth muscle contractility, alterations in the enteric nervous system function, and accumulation of glycosaminoglycans within the bowel wall [1]. Although constipation is commonly observed in hypothyroid patients, severe intestinal obstruction remains an uncommon presentation and may delay diagnosis [2]. We present a rare case of autoimmune hypothyroidism complicated by fecaloma, suspected stercoral colitis, and subacute intestinal obstruction.

## Case presentation

A 41-year-old male patient with diabetes mellitus presented with abdominal distension, constipation, and metabolic derangement examination. Clinical examination revealed evidence of dehydration, with dry, rough skin and vitiligo over the abdomen. The abdomen was distended, with abdominal girth ranging from 105 to 118 cm, and bowel sounds were sluggish. He was initially diagnosed with diabetic ketoacidosis (DKA) accompanied by paralytic ileus secondary to severe hypokalemia.

The patient received standard DKA management, including intravenous fluid resuscitation, insulin therapy, and electrolyte replacement. Following treatment, the metabolic acidosis and ketoacidosis resolved. However, persistent constipation and progressive abdominal distension continued despite correction of electrolyte abnormalities.

Multiple conservative bowel decompression measures were attempted, including enemas and rectal tube insertion, with minimal clinical response. Due to ongoing symptoms and worsening abdominal distension, endoscopic decompression with rectal tube placement was performed. Following the procedure, the patient passed a large volume of stool; however, significant abdominal distension persisted.

Further clinical course

Nasogastric tube suction and rectal tube decompression were maintained for 24 hours. Despite these interventions, abdominal distension persisted. During hospitalization, the patient developed severe hypernatremia (serum sodium approximately 150 mmol/L) accompanied by elevated blood pressure readings but he was not hypertensive before.

**Figure 1:**
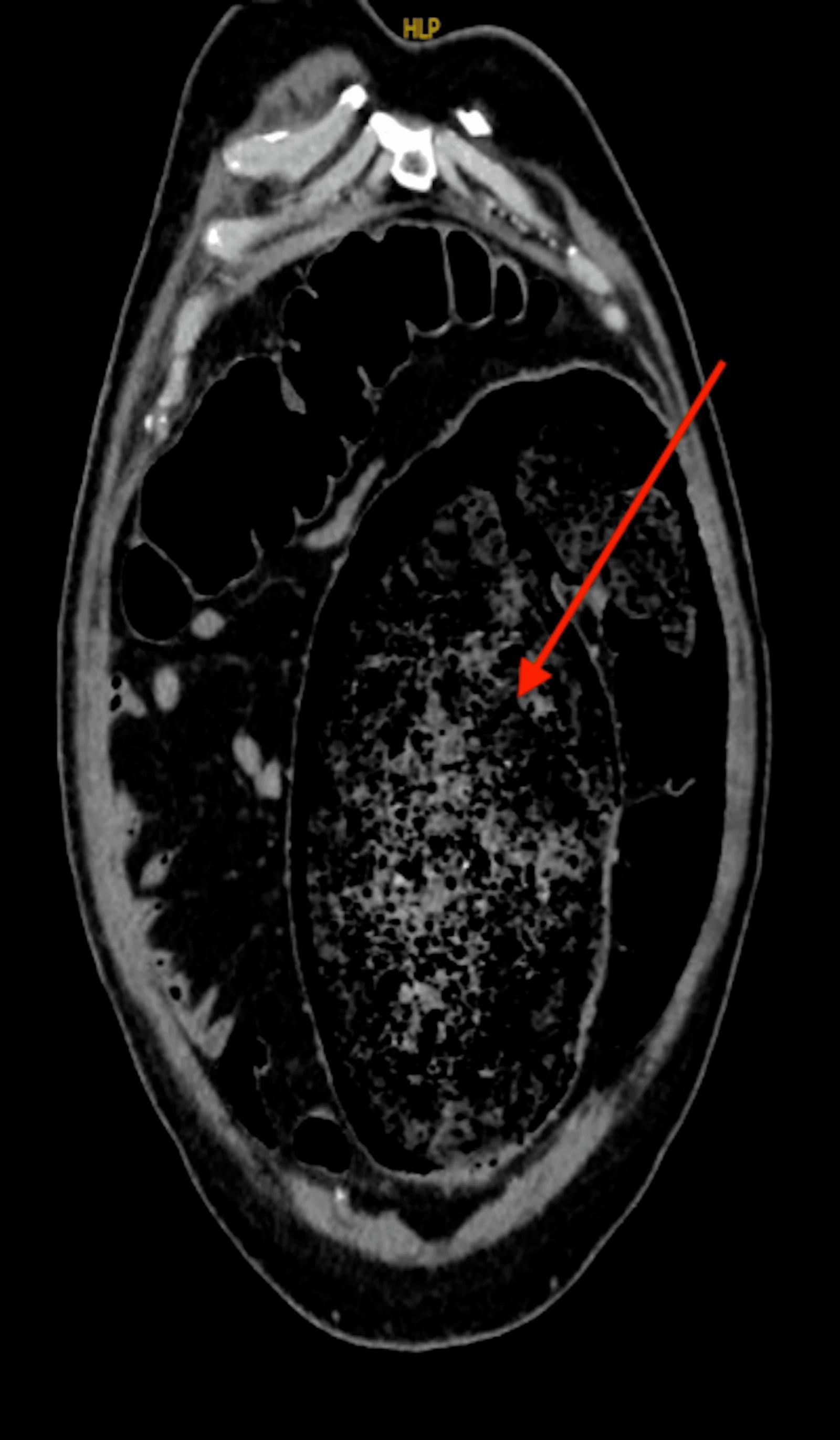
Axial contrast-enhanced compted tomography (CT) abdomen CT abdomen showing a large sigmoid fecaloma (red arrow) causing marked colonic dilatation and ileus, with associated features suggestive of stercoral colitis in a patient with severe hypothyroidism-induced gastrointestinal hypomotility.

**Figure 2:**
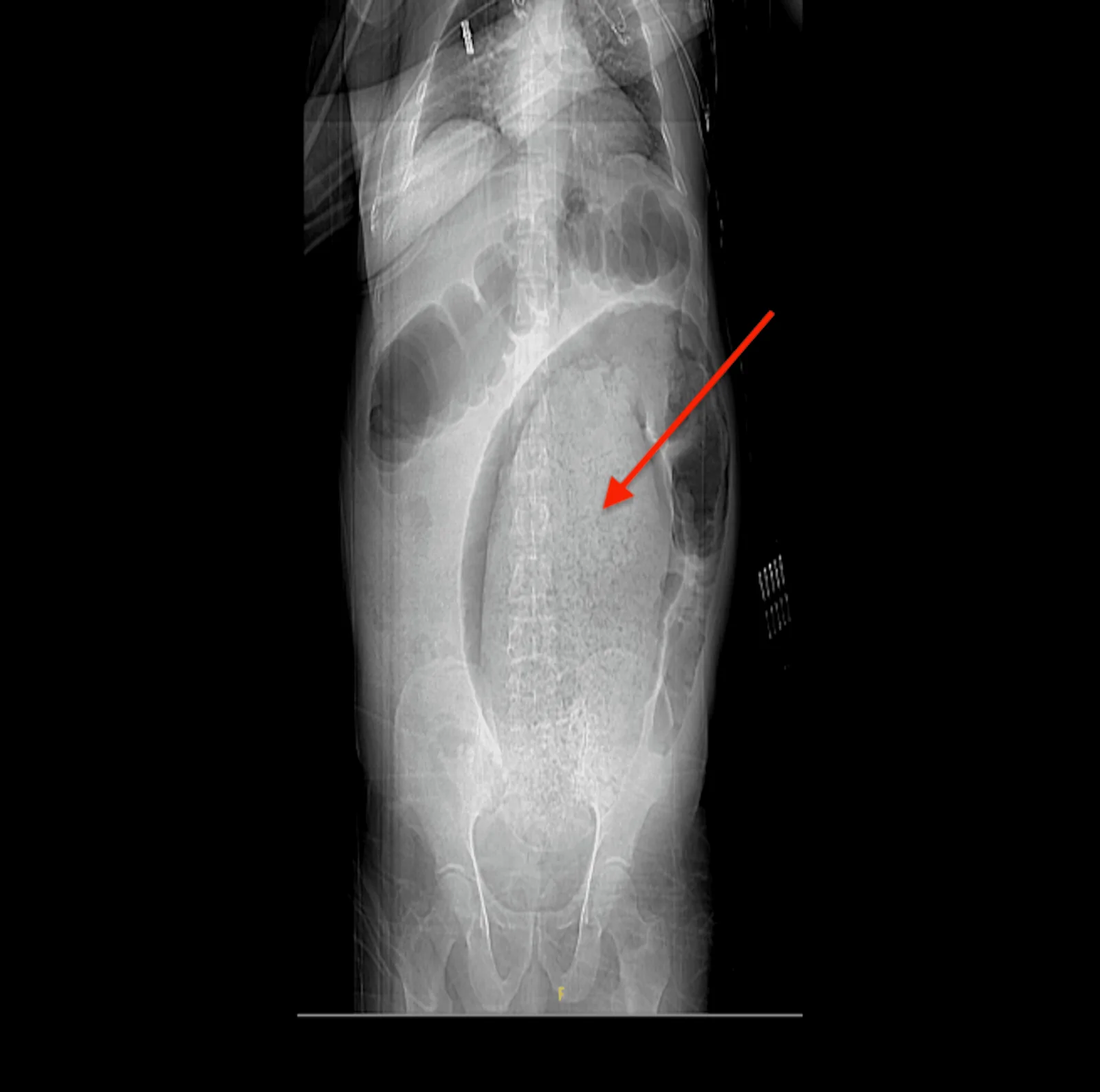
Plain X-ray abdomen Plain abdominal radiograph demonstrating a giant rectosigmoid fecaloma (red arrow) with marked diffuse gaseous distension of the colon, consistent with colonic ileus and subacute large bowel obstruction.

**Figure 3:**
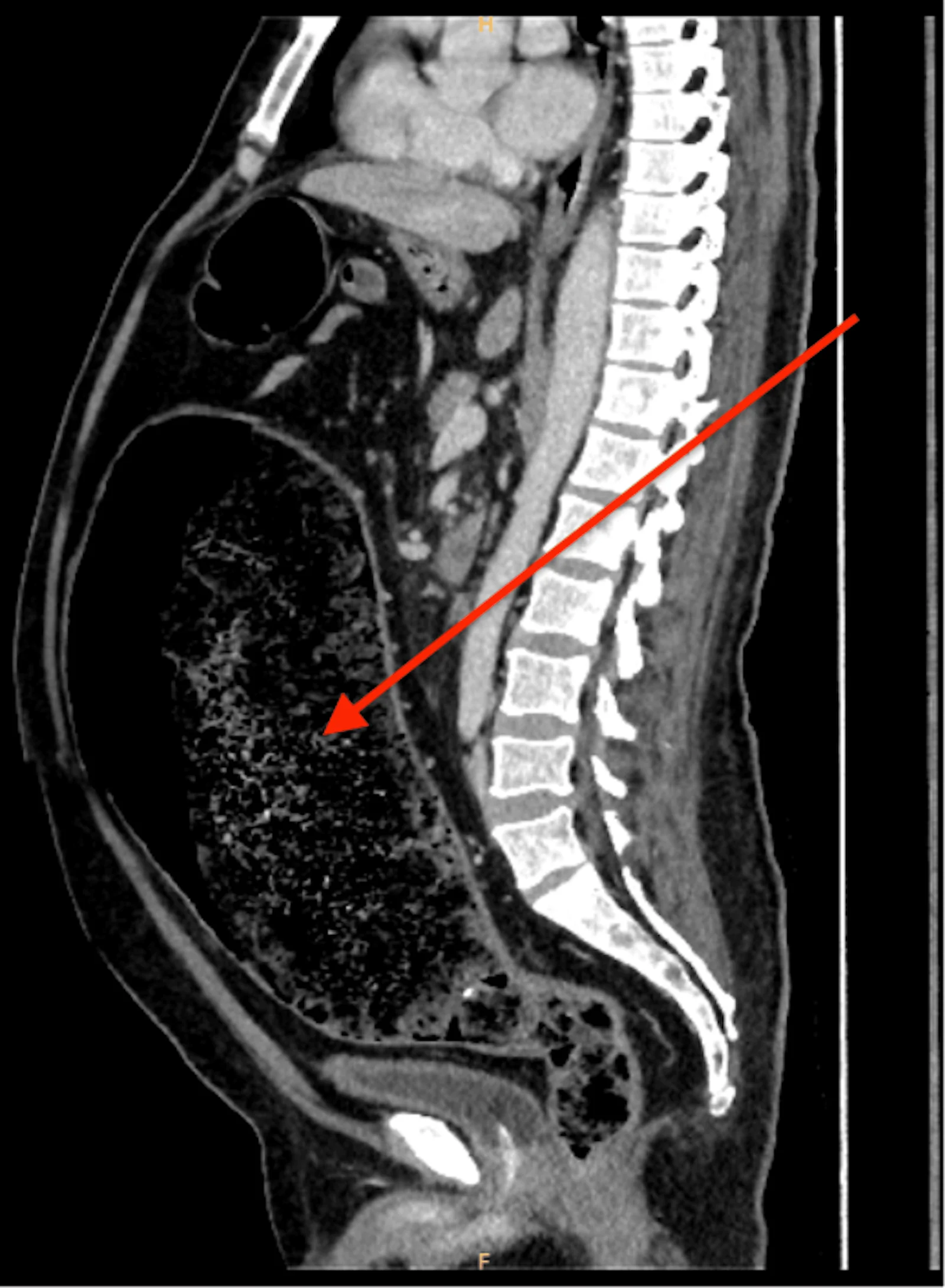
Computed tomography (CT) abdomen - sagittal section Sagittal contrast-enhanced CT abdomen showing an extensive rectosigmoid fecaloma (red arrow) occupying the dilated colon with associated diffuse colonic dilatation and ileus, consistent with severe fecal impaction.

Radiological findings are presented in Table 1 and Figures 1-3.

**Table 1:** Computed Tomography Abdomen Findings and Clinical Significance

Radiological Finding	Clinical Interpretation
Marked diffuse gaseous distension of the colon	Significant colonic dilatation secondary to impaired bowel motility
Severe fecal loading within the sigmoid colon with fecaloma formation	Severe fecal impaction contributing to mechanical obstruction-like features
Features suggestive of stercoral colitis	Inflammatory changes due to prolonged fecal retention and pressure effect
Associated colonic ileus	Functional bowel hypomotility contributing to subacute intestinal obstruction
Mild hepatosplenomegaly	Additional radiological finding
Minimal ascites	Small-volume intra-abdominal free fluid

Further assessment revealed clinical features suggestive of autoimmune polyglandular syndrome, as mentioned in Table 2.

**Table 2:** Clinical and biochemical evaluation revealing severe autoimmune hypothyroidism in the context of autoimmune features, supporting a diagnosis of autoimmune polyglandular syndrome. TSH: Thyroid-stimulating hormone; TPO: thyroid peroxidase.

Category	Findings
Clinical features	Diabetes mellitus
	Vitiligo
	Dry skin
	Severe constipation with persistent colonic hypomotility
Electrolyte abnormalities	Persistent hypokalaemia (ranging from 2.57 mEq/L to 3.5 mEq/L after correction)
	Hypernatremia (serum sodium approximately 150 mmol/L)
Cardiovascular findings	Elevated blood pressure readings during admission
Thyroid function	Markedly elevated TSH: 32 mIU/L
Thyroid autoimmunity	Positive thyroid autoantibodies consistent with autoimmune thyroiditis (TPO more than 400 mIU and thyroglobulin antibody more than 1000 mIU )
Renin-aldosterone profile	Suppressed plasma renin activity (<2.5 ng/l)
	Low aldosterone concentration

Based on these findings, severe autoimmune hypothyroidism was diagnosed. Levothyroxine replacement therapy was initiated. Simultaneously, bowel preparation with polyethylene glycol solution (Clean-Prep®) was administered in anticipation of possible colonoscopic evaluation.

Within 48 hours of initiating thyroid hormone replacement, gradual improvement in bowel motility was observed with the passage of stool and reduction in abdominal distension. Repeat laboratory investigations demonstrated correction of electrolyte abnormalities. Nasogastric and rectal tubes were subsequently removed.

After one week, the patient's clinical condition improved significantly, allowing transfer from intensive care to a general medical ward. Levothyroxine dosage was optimized, and the patient was discharged home with outpatient endocrine follow-up.

## Discussion

This case highlights an uncommon but clinically important presentation of autoimmune hypothyroidism manifesting as persistent colonic hypomotility complicated by a giant faecaloma, suspected stercoral colitis, and subacute intestinal obstruction. Although constipation is a common gastrointestinal manifestation of hypothyroidism, progression to paralytic ileus, intestinal pseudo-obstruction, or bowel obstruction-like presentations is rare and may delay the recognition of the underlying endocrine disorder [1,2].

In our patient, the diagnosis was initially challenging due to concurrent DKA and significant electrolyte abnormalities, particularly hypokalaemia, which are both recognised contributors to impaired gastrointestinal motility. However, persistent abdominal distension and faecal loading despite correction of metabolic abnormalities suggested an additional reversible cause of bowel dysmotility.

Thyroid hormones play an important role in maintaining normal gastrointestinal function through effects on smooth muscle activity, autonomic regulation, and enteric nervous system signalling. Severe thyroid hormone deficiency reduces intestinal motility and may lead to tissue changes associated with mucopolysaccharide accumulation, contributing to marked bowel hypomotility [3].

In addition, diabetic autonomic neuropathy may have contributed to impaired colonic transit in this patient. Although hypokalaemia is a recognised reversible cause of ileus because of impaired neuromuscular function, the persistence of bowel dysmotility following electrolyte correction suggested that hypothyroidism represented the principal underlying pathology [4].

Only a limited number of cases describing hypothyroidism presenting as intestinal obstruction have been reported. Kubiszewski et al. reported a case of uncontrolled Hashimoto thyroiditis causing radiological features suggestive of bowel obstruction without a mechanical cause, with clinical improvement following levothyroxine replacement [5].

Similar to that report, our patient underwent extensive gastrointestinal investigations and bowel decompression before autoimmune hypothyroidism was recognised as the underlying reversible cause. This case further demonstrates that thyroid dysfunction should be considered in patients with persistent bowel dysmotility despite correction of more common metabolic abnormalities. A distinctive feature of this case was the presence of a large faecaloma with CT findings suggestive of stercoral colitis. Stercoral colitis results from prolonged faecal impaction causing pressure-related colonic inflammation, ulceration, ischemia, and potentially perforation. Early recognition on CT imaging is essential to guide timely bowel decompression and prevent complications [6]. In this patient, the absence of perforation allowed successful conservative and endoscopic management alongside thyroid hormone replacement.

The coexistence of autoimmune thyroiditis, diabetes mellitus, and vitiligo also raised the possibility of autoimmune polyglandular syndrome (APS). APS comprises a group of disorders characterised by the coexistence of multiple autoimmune endocrine diseases. APS type 2 is classically defined by the presence of autoimmune Addison's disease together with autoimmune thyroid disease and/or type 1 diabetes mellitus, whereas APS type 3 describes autoimmune thyroid disease occurring with other autoimmune disorders, such as type 1 diabetes mellitus or vitiligo, in the absence of adrenal insufficiency [7].

In our patient, strongly positive anti-thyroid peroxidase and anti-thyroglobulin antibodies confirmed autoimmune thyroiditis, while diabetes mellitus and vitiligo suggested autoimmune clustering. However, the absence of biochemical evidence of primary adrenal insufficiency, together with suppressed plasma renin activity and low aldosterone concentrations, did not support a diagnosis of APS type 2. Consequently, this patient was considered to have features suggestive of autoimmune polyglandular disease requiring ongoing endocrine surveillance rather than a confirmed diagnosis of APS. Further assessment, including adrenal autoantibody testing and periodic evaluation of adrenal function, would be appropriate given the increased risk of developing additional autoimmune endocrinopathies [7].

This case emphasises that persistent ileus or bowel obstruction should not always be attributed solely to DKA, electrolyte disturbances, or simple constipation. Thyroid function testing is an inexpensive and valuable investigation in patients with unexplained gastrointestinal hypomotility, particularly when accompanied by clinical features suggestive of hypothyroidism or other autoimmune diseases. Early recognition of autoimmune hypothyroidism allows prompt initiation of levothyroxine replacement, may prevent unnecessary invasive procedures, and can lead to rapid recovery when combined with appropriate bowel decompression and supportive management.

## Conclusions

Autoimmune hypothyroidism is an uncommon but important reversible cause of severe gastrointestinal dysmotility and subacute intestinal obstruction. In patients presenting with persistent constipation, abdominal distension, faecaloma, ileus, or pseudo-obstruction without an identifiable mechanical cause, thyroid dysfunction should be considered as part of the diagnostic evaluation, particularly when gastrointestinal symptoms persist despite correction of metabolic abnormalities. Early recognition and prompt initiation of levothyroxine replacement, together with appropriate bowel decompression, may prevent unnecessary invasive procedures and result in significant clinical improvement. This case also highlights the importance of recognising coexisting autoimmune diseases and considering the possibility of an underlying autoimmune polyglandular syndrome or autoimmune predisposition, with appropriate endocrine evaluation and long-term follow-up where clinically indicated.
